# Supplementary material for: Clustering and Healthcare Costs With Multiple Chronic Conditions in a US Study
Source: Front Public Health. 2021 Jan 21;8:607528. doi: 10.3389/fpubh.2020.607528 (PMC7859629; doi:10.3389/fpubh.2020.607528)
Supplement: Supplementary file 1 [file Data_Sheet_1.PDF]

Technical Supplement

Definitions for Healthcare Spending

Healthcare spending included financial claims for all healthcare episodes and definitions for healthcare spending and type of service delivery as below:

Total Cost – total of reimbursed drug and medical costs

Medical Cost – total reimbursed medical costs (also known as medical benefit)

Inpatient Cost – total net reimbursed of inpatient services

Outpatient Cost – total net reimbursed for outpatient services

Speciality Procedure and Diagnostics – total reimbursed for speciality procedures

Primary Care Services – total reimbursed for primary care procedures

Psychiatric Services – total reimbursed for psychiatric service procedures

Emergency Services - total reimbursed for emergency services

Drug cost – total net reimbursed drug cost (also known as pharmacy benefit)

Generic drug cost – total net reimbursed drug cost for drugs categorized as generics

Branded drug cost – total net reimbursed drug cost for branded drugs

Copay – patient out of pocket drug cost

Number of conditions	Number of patients with conditions	Average cost per patient	Number of **CCAE** patients with conditions	Average cost per **CCAE** patient	Number of **Medicaid** Patients with conditions	Average cost per **Medicaid** patient	Number of **Medicare** patients with conditions	Average cost per **Medicare** patient
2	23,551,408	$ 4,385	18,464,155	$ 4,532	2,231,793	$ 3,555	2,855,461	$ 4,083
3	20,166,132	$ 5,851	14,520,758	$ 6,099	1,813,055	$ 4,935	3,832,319	$ 5,343
4	16,392,071	$ 7,735	10,535,366	$ 8,241	1,511,182	$ 6,439	4,345,523	$ 6,960
5	12,804,515	$ 10,088	7,248,729	$ 11,070	1,245,461	$ 7,987	4,310,326	$ 9,045
6	9,762,840	$ 12,739	4,849,420	$ 14,117	1,031,158	$ 10,256	3,882,263	$ 11,676
7	7,430,641	$ 16,247	3,187,880	$ 18,525	853,450	$ 12,447	3,389,311	$ 15,061
8	5,611,530	$ 19,856	2,059,279	$ 23,305	695,610	$ 14,674	2,856,641	$ 18,632
9	4,290,681	$ 24,181	1,353,481	$ 29,509	555,028	$ 16,920	2,382,172	$ 22,846
10	3,194,766	$ 28,646	840,456	$ 36,379	451,129	$ 19,772	1,903,182	$ 27,335
11	2,448,914	$ 33,874	555,969	$ 44,098	360,018	$ 22,540	1,532,927	$ 32,827

Table 1. Comparison of health spending between insurance type, by number of conditions The data are scaled to the total US Adult 2015 population

	Condition 1	Condition 2	Patient Count (%)
1	HIGH_CHOLESTEROL	HYPERTENSION	281,901 (30.77 %)
2	DIABETES	HYPERTENSION	151,195 (16.23 %)
3	HYPERTENSION	OSTEOARTHRITIS	103,129 (11.07 %)
4	HIGH_CHOLESTEROL	OTHER_ENDOCRINE	101,947 (10.94 %)
5	EYE_PROBLEM	HIGH_CHOLESTEROL	96,062 (10.31 %)
6	HYPERTENSION	OTH_NEURO_DIS	95,260 (10.23%)
7	HYPERTENSION	PUD_EXCLUDE_BLEED	93,537 (10.04 %)
8	ANXIETY	DEPRESSION	89,326 (9.59 %)
9	HIGH_CHOLESTEROL	WEIGHT_LOSS	87,657 (9.41 %)
10	ALLERGY	HYPERTENSION	87,293 (9.37 %)
11	DEPRESSION	HYPERTENSION	84,388 (9.06 %)
12	ALLERGY	HIGH_CHOLESTEROL	79,210 (8.50 %)
13	HYPERTENSION	OBESITY	79,018 (8.48 %)
14	CAD	HYPERTENSION	78,890 (8.47 %)
15	CARDIAC_ARRH	HYPERTENSION	74,290 (7.97 %)
16	DEPRESSION	HIGH_CHOLESTEROL	68,671 (7.37 %)
17	ANXIETY	HIGH_CHOLESTEROL	61,369 (6.59 %)
18	CARDIOMYOPATHY_HEART_DISEASE	HYPERTENSION	61,880 (6.46 %)
19	CARDIOMYOPATHY_HEART_DISEASE	HIGH_CHOLESTEROL	51,295 (5.50 %)
20	DIABETES	OTHER_ENDOCRINE	49,758 (5.344 %)
21	DIABETES	OTH_NEURO_DIS	49,455 (5.31 %)
22	HYPERTENSION	RENAL_FAILURE	44,353 (4.76 %)
23	EYE_PROBLEM	OTHER_ENDOCRINE	41,685 (4.477 %)
24	HYPERTENSION	MALIGNANT_NEOPLASM	41,754 (4.48 %)

25	ALLERGY	EYE_PROBLEM	40,771 (4.37 %)

Table 2. Top 25 Cluster Pairs of Conditions according to Prevalence

	Condition 1	Condition 2	Patient Count (%)
1	HYPERTENSION	HIGH_CHOLESTEROL	281,901 (30.28 %)
2	DIABETES	HYPERTENSION	151,195 (16.24 %)
3	DIABETES	HIGH_CHOLESTEROL	133,956 (14.39 %)
4	OSTEOARTHRITIS	HYPERTENSION	103,129 (11.08 %)
5	DEPRESSION	ANXIETY	89,326 (9.59 %)
6	HYPERTENSION	CAD	78,890 (8.47 %)
7	HIGH_CHOLESTEROL	CAD	70,624 (7.59 %)
8	HIGH_CHOLESTEROL	ANXIETY	61,369 (6.59 %)
9	OTH_NEURO_DIS	CHRONIC_PAIN	59,003 (6.34 %)
10	COPD	ALLERGY	44,486 (4.78 %)
11	HYPERTENSION	RENAL_FAILURE	44,353 (4.76 %)
12	DIABETES	CAD	37,264 (4.00 %)
13	COAGULOPATHY	BLOOD_LOSS_ANEMIA	37,943 (4.08 %)
14	CARDIAC_ARRH	CARDIOMYOPATHY_HEART_DISEASE	31,279 (3.36 %)
15	COPD	CAD	26,633 (2.86 %)
16	CARDIAC_ARRH	VALVULAR	24,386 (2.62 %)
17	DEPRESSION	CHRONIC_PAIN	23,897 (2.57 %)
18	DIABETES	RENAL_FAILURE	24,710 (2.65 %)
19	ANXIETY	CHRONIC_PAIN	22,841 (2.45 %)
20	OSTEOARTHRITIS	CHRONIC_PAIN	20,897 (2.24 %)
21	COPD	CHRONIC_PAIN	18,225 (1.96 %)
22	RENAL_FAILURE	CAD	15,656 (1.68 %)

23	RENAL_FAILURE	BLOOD_LOSS_ANEMIA	13,403 (1.44 %)
24	CHF	RENAL_FAILURE	13,256 (1.42 %)
25	WEIGHT_LOSS	CYSTIC_FIBROSIS	8,983 (0.96 %)

Table 3. Top 25 Cluster Pairs of Conditions according to Strength of Association

	Conditions with Strong Associations *(>95%)	Conditions with Moderate Associations *(15-95%)	Patient Count (%)
1	Hypertension<br>High Cholesterol	Other Endocrine<br>Eye Problems	123,203 (13.23%)
2	High Cholesterol	Other Endocrine<br>Weight Loss<br>Allergy	106,549 (11.44%)
3	Hypertension	Diabetes<br>Allergy	96,023 (10.31%)
4	Depression	Anxiety<br>Alcohol Abuse	85,184 (9.15%)
5	Diabetes<br>High Cholesterol<br>Hypertension	Eye Problems<br>Other Endocrine<br>Obesity	73,935 (7.94%)
6	Other Endocrine	Hypertension<br>Weight Loss	62,950 (6.76%)
	Peptic Ulcer Disease Excluding Bleed	Hypertension<br>Allergy	58,446 (6.23%)
8	Hypertension<br>Coronary Artery Disease<br>Cardiomyopathy	Cardiac Arrythmia<br>Diabetes<br>Eye Problems	54,591 (5.86%)
9	Other Neurological Disorder<br>Chronic Pain	Back Pain<br>Alcohol Abuse	42,121 (4.52%)
10	Hypertension<br>High Cholesterol<br>Other Neurological Disease<br>Depression	COPD<br>Peptic Ulcer Disease Excluding Bleed<br>Diabetes<br>Back Pain<br>Other Endocrine Disease	40,266 (4.32%)

	Osteoarthritis<br>Anxiety		

Table 4. K-Means Clustering of 2 or more Conditions *The second column reports the main chronic conditions that were present in over 95% of patients falling within that cluster; column three reports conditions that were present in 20-95% of patients within that cluster
